# Evaluating the effect of an in-service training workshop on ICD-10 coding instructions of pregnancy, childbirth and the puerperium for clinical coders

**DOI:** 10.25122/jml-2021-0062

**Published:** 2021

**Authors:** Somayeh Paydar, Farkhondeh Asadi

**Affiliations:** 1. Department of Health Information Technology, School of Paramedical Sciences, Kermanshah University of Medical Sciences, Kermanshah, Iran; 2. Department of Health Information Technology and Management, School of Allied Medical Sciences, Shahid Beheshti University of Medical Sciences, Tehran, Iran

**Keywords:** clinical coders, evaluation, ICD-10, in-service training, Kirkpatrick model

## Abstract

The quality of the data coded based on the 10^th^ revision of the International Classification of Diseases (ICD-10) can be improved by providing continuous education and promoting the clinical coders’ knowledge and skills. Due to the significance of maternal health in promoting the health of society, the present study evaluated the effects of an in-service training workshop on ICD-10 coding instructions of pregnancy, childbirth, and the puerperium for clinical coders. This applied evaluation study was conducted to evaluate the effects of a coding instructions training course focusing on the 15^th^ chapter of the ICD-10. The statistical population comprised 45 clinical coders working in the hospitals. The data were collected by a researcher-made questionnaire scored on a five-point Likert scale at the reaction level and by pretest and posttest questionnaires at the learning level. The data were then analyzed by descriptive statistics at the reaction level and by a paired-samples t-test at the learning level. The participants’ satisfaction with the training course was 94.7% on average at the reaction level. At the learning level, the results of the paired-samples t-test showed a significant difference between the means of scores before and after the training course (p=0.000). The training course led to satisfaction and enhanced the capabilities of the clinical coders with regard to coding the 15^th^ chapter of ICD-10. Clinical Coders must receive training on the new changes and guidelines in the other chapters of ICD-10 based on its most recent revision and employ them in the workplace.

## Introduction

Maternal health refers to women’s health during pregnancy, delivery, and the postpartum period [1]. The World Health Organization (WHO) states that 810 women die every day due to complications of pregnancy and delivery [2]. Severe hemorrhage following delivery, infections (often post-delivery), high blood pressure during pregnancy (pre-eclampsia and eclampsia), and the complications of delivery and unsafe abortions are the most important complications accounting for 75% of the cases of maternal mortality. The majority of pregnancy- and delivery-related complications can be prevented and treated [2, 3]. Maternal mortality is a key and reliable qualitative index of national and economic development, affected by women’s literacy, communication tools, access to healthcare and midwifery emergency services, medical expenses, household income, and other factors [3].

For monitoring the complications of pregnancy and delivery, all the diagnoses related to pre-pregnancy risk factors, diseases, complications, and interventions during pregnancy and delivery are coded based on the International Statistical Classification of Diseases and Related Health Problems, 10^th^ Revision (ICD-10), and then these codes are recorded in healthcare systems and national registries [4]. The main goal of coding the data based on ICD-10 is to provide an accurate, consistent, and brief view of the information related to patients’ states and the services offered to them [5]. ICD-10 is used to code the causes of mortality recorded in death certificates and hospitalization periods, monitor mortality and morbidity, create disease registries, evaluate healthcare policies, and provide healthcare financial resources [4, 6, 7]. This classification is revised and updated by the WHO [4]. To enjoy the benefits of ICD-10, the accurate coding of diseases and the quality of the codes allocated to clinical data in healthcare are of utmost importance. They are directly related to patient care, income, and performance evaluation [7]. The use of high-quality coded data improves the quality of services, ensures equitable healthcare reimbursement, and helps researchers conduct high-quality research [5]. Without high-quality coded data, healthcare workers cannot make optimal decisions for patient care [8].

The quality of the coded data is affected by two major factors: first, the extent to which healthcare providers (primarily the physicians) document the treatments and diagnoses in the patients’ health records precisely, completely, and clearly; and second, the extent to which the health records are coded by healthcare clinical coders accurately and consistently [9]. According to Alonso, the main obstacles to ensuring the quality of coded data are the clinical coders’ limited understanding of the medical terminology, the clinical coders’ experience, or problems within health records, e.g., a lack of specificity of the recorded data or imperfections of the classification system [10]. Clinical Coders are responsible for the accuracy, completeness, and timeliness of the allocated codes, factors that ensure the quality of coding [11].

The accurate and reliable coding of patient information is essential for clinical coders because any mistake can lead to an inaccurate representation of the patient care episode and billing mistakes [8, 12]. The low reliability of the codes is the result of insufficient training and standardization for the clinical coders [13], and coding quality can be improved by providing continuing education and enhancing the clinical coders’ communication with healthcare workers [9]. Clinical coders’ knowledge and skills can be expanded by updating their medical knowledge, holding training courses, workshops, seminars, and performing clinical coder accreditation. Wide-ranging and continuing education for clinical coders is necessary because of the importance of the quality of codes in predicting the budget, creating electronic health records, and conducting research [14].

One way to update and improve the knowledge and skills of healthcare workers, including clinical coders, is to provide in-service training [15–17]. Professional coder training is a fundamental method for eliminating code inconsistency [11]. Since clinical coders generate the data and are the key determinants of the quality of ICD codes, in-service training is essential for them [18].

High-quality and accurate data are essential due to the significance of maternal health in promoting the health of society and the necessity of continuous monitoring of their problems during pregnancy, delivery, and the postpartum period. The requirement of having access to these data is the accurate, precise, and complete coding of the data by clinical coders. Developing training programs on obstetrics/gynecology coding for coding specialists by using ICD-10 codes is critical for coding quality promotion. Therefore, the present study evaluated the effects of an in-service training workshop on ICD-10 coding instructions of pregnancy, childbirth, and the puerperium for clinical coders in hospitals affiliated with Shahid Beheshti University of Medical Sciences (Tehran, Iran).

## Material and Methods

This applied evaluation study was conducted to evaluate the effects of a coding instructions training course focusing on the 15^th^ chapter (pregnancy, childbirth and the puerperium) of the ICD-10.

The statistical population comprised the clinical coders working in the mentioned hospitals (n=45). As one of the best evaluation methods, the Kirkpatrick evaluation model was adopted to assess whether the training program met the needs of the learners [15, 19]. In this model, the evaluation process is divided into four levels of reaction, learning, behavior, and results. Kirkpatrick defines the reaction level as a measure of the customers’ or learners’ satisfaction. The second level, learning, measures the increased knowledge or capability after the training. In the third level, behavior refers to the extent and nature of changes in the participants’ behavior as a result of the training course. The fourth level, results, specifies the extent to which the objectives set by the organization are achieved following the training course [20–23]. Some studies have focused on the second level of this model for examining talent development, while others have investigated the third and fourth levels as training interventions. Still, most of the studies using the Kirkpatrick model have evaluated only the first and second levels [19]. Likewise, the present study used two levels of reaction and learning of the said model.

At the reaction level, the data were collected via a researcher-made questionnaire based on the learner satisfaction components of the Kirkpatrick model. The questionnaire included five closed-ended questions (scored on a Likert scale) to measure the participants’ satisfaction. The answers were strongly disagreeing, somewhat disagree, neither agree nor disagree, somewhat agree, and strongly agree, which received scores of 1 to 5, respectively. The sum of the “somewhat agree” and “completely agree” scores indicated an optimal satisfaction level. The content validity of the questionnaires was assessed, and their reliability was checked via Cronbach’s alpha.

The level of learning was evaluated by a pretest and a posttest. A questionnaire with ten questions was developed and administered before and after the training course. The content validity of the questionnaires was assessed, and their reliability was examined by the test-retest method. At the reaction level, the data were analyzed in Excel via descriptive statistics (number and percentage). At the learning level, the data distribution was normal, based on the Kolmogorov-Smirnov test; the pretest and posttest mean scores were compared via a paired-samples t-test. The data were analyzed using the Statistical Package for the Social Sciences (SPSS) version 22, and the significance level of 0.05 was set for the paired-samples t-test.

## Results

The results are divided into two levels of reaction and learning.

### Reaction level (the participants’ satisfaction level):

Based on Table 1, 95.6% of the clinical coders believed that participation in the in-service training course had increased their knowledge and awareness of coding instructions regarding the 15^th^ chapter of ICD-10. As for the teaching method, 91.1% of the participants deemed the presentation of the content by the instructor to be appropriate. Moreover, 97.8% of the participants believed that the presented content was relevant to their job as clinical coders. Furthermore, 91.1% were generally satisfied with the course, and no participants were dissatisfied with the course. Finally, 97.8% of the participants were willing to recommend the in-service training course to other clinical coders.

**Table 1.** The frequency distribution of the participants’ satisfaction with the in-service training workshop on ICD-10 coding instructions of pregnancy, childbirth and the puerperium.

**Criterion**	**Strongly Disagree**	**Somewhat Disagree**	**Neither Agree nor Disagree**	**Somewhat Agree**	**Strongly Agree**
	**No. (%)**	**No. (%)**	**No. (%)**	**No. (%)**	**No. (%)**
**This course improved my knowledge and awareness of the topic.**	0 (0)	0 (0)	2 (4.4)	10 (22.2)	33 (733.3)
**The expression and presentation of the content by the instructor (the method of instruction) were appropriate.**	0 (0)	0 (0)	4 (8.9)	9 (20)	32 (71.1)
**The content presented in this course was relevant to my job.**	0 (0)	0 (0)	1 (2.2)	2 (4.4)	42 (93.3)
**I was generally satisfied with the course.**	0 (0)	0 (0)	4 (8.9)	8 (17.8)	33 (73.3)
**I will recommend this course to others.**	0 (0)	0 (0)	1 (2.2)	7 (15.6)	37 (82.2)

### Learning level

Table 2 presents descriptive indices, including the mean and standard deviation (SD) of the two groups on the pretest and posttest for the learning level.

**Table 2.** The mean and SD of pre- and posttest scores of the 15^th^ chapter of ICD-10 coding instruction.

**Group**	**No.**	**Mean**	**Std. Deviation**
**Pretest**	45	3.53	2.085
**Posttest**	45	8.40	1.232

The paired-samples t-test showed a significant difference between the mean scores before and after the training course (p=0.000), indicating that this course improved the participants’ level of learning (Table 3).

**Table 3.** The results of the paired-samples t-test of the participants’ learning level.

**Learning Level**		**Paired Differences**		**t**	**df**	**Sig. (2-tailed)**
		**Mean**	**Std. Deviation**			
**Pair**	Pretest – posttest	-4.867	1.471	-22.195	44	.000

## Discussion

This study evaluated the effects of an in-service training course for clinical coders about the 15^th^ chapter (pregnancy, delivery, and the puerperium) of the ICD-10 (2016 edition). This evaluation was performed based on the first (reaction) and second (learning) levels of the Kirkpatrick model. The results at the reaction level showed that holding the in-service training course enhanced the clinical coders’ awareness of the guidelines of the 15^th^ chapter of ICD-10. Overall, the majority of the participants were satisfied with the course. According to Doktorchik *et al.*, the ICD system is adopted for coding hospital data (admission, emergency department visits, and daily surgeries) so that the resulting information would be used for research and reporting. Therefore, it is essential that clinical coding is of high quality so that accurate healthcare data can be prepared for patient safety purposes, healthcare quality assessment, research, monitoring, hospital management, and resource allocation [24]. The results of the study conducted by Alipour and Ahmadi also indicated that one way to improve the quality of coding is by holding training courses for clinical coders on the new changes in the revised edition of coding books and the regulations related to different chapters of these books [25]. In Iran, no study had been conducted to evaluate the effectiveness of in-service training for clinical coders based on the Kirkpatrick model.

Similar to this study, Li *et al.* evaluated a training program for nurses working in emergency surgery departments during the COVID-19 pandemic. The reaction-level results revealed that the nurses were highly satisfied with the educational content, the training length, and the applications of this course in clinical work [26]. Oashttamadea has also noted the significant role of training in improving the accuracy of delivery coding [11].

Consistent with the present study, the results reported by Yi *et al.* [27], Tahmasebi *et al.* [28], Walker *et al.* [29], Piryani *et al.* [30], and Mohan *et al.* [31] also demonstrated that participants were highly satisfied with the training programs in the dimensions of content, instructor, method of instruction, facilities, and equipment.

As for the second level of the Kirkpatrick model, the findings showed that this course made the clinical coders more familiar with the new instructions and changes in the 15^th^ chapter of ICD-10 and enhanced their learning level of this chapter. Furthermore, Santos *et al.* referred to a lack of continuing education for clinical coders as an organizational factor impacting the accuracy, completeness, and timeliness of coding and noted that the quality of the codes could be improved by providing training for the clinical coders [16].

Li *et al.*’s evaluation of theoretical and operational exams of the nurses indicated that the participants’ scores at the learning level were significantly increased after the course (p<0.001) [26]. Moreover, Yoon *et al.* evaluated a continuing education program based on levels 1, 2, and 3 of the Kirkpatrick model for the professional development of physicians and physician assistants in Laos hospitals. Their results demonstrated the high satisfaction and progress of the participants. In their study, the participants employed the knowledge and skills acquired during the course in their profession and even instructed their colleagues. This, in turn, led to considerable improvement in central, provincial, and district hospitals [32]. Also, in the studies conducted by Cullinante *et al.* [20] and Mohan *et al.* [31], a significant difference in the learning level was observed before and after the training course, which is consistent with the findings of the present study.

Some factors that enhanced learning after the course in this study could have been the clinical coders’ motivation, their need for such courses, and the relevance of the instructed content to their educational needs. Moreover, the merits of the course were the participation of the clinical coders in theoretical and practical topics and the practical nature of the content.

## Conclusion

This study revealed the personal and organizational benefits of in-service training for clinical coders and demonstrated the clinical coders’ interest in such courses. Therefore, the Ministry of Health and Medical Education and the Deputy for Treatment of universities should provide continuing in-service training programs for clinical coders based on the most recent revision of ICD-10.

## Acknowledgments

The authors wish to express gratitude to the Vice-Chancellor in Treatment Affairs of the Shahid Beheshti University of Medical Sciences for holding this workshop.

### Ethical approval

The approval for this study was obtained from the Ethics Committee of the Shahid Beheshti University of Medical Sciences (approval ID: IR.SBMU.RETECH.REC.1399.887.

### Consent to participate

The participants were assured that the completed questionnaire would be anonymous and confidential and gave their consent for participating in this study.

### Conflict of interest

The authors declare that there is no conflict of interest.
